# Does Quality Certification Work? An Assessment of Manyata, a Childbirth Quality Program in India’s Private Sector

**DOI:** 10.9745/GHSP-D-22-00093

**Published:** 2022-12-21

**Authors:** Megan Marx Delaney, Gulnoza Usmanova, Tapas Sadasivan Nair, Vanessa L. Neergheen, Kate Miller, Eliza Fishman, Nitin Bajpai, Parvez Memon, Lauren Bobanski, Dinesh Singh, Vineet Kumar Srivastava, Hema Divakar, Hrishikesh Pai, Katherine E. A. Semrau, Suranjeen Prasad Pallipamula

**Affiliations:** a Ariadne Labs, a joint center of the Harvard T.H. Chan School of Public Health and Brigham and Women’s Hospital, Boston, MA, USA.; b Jhpiego India Country Office, New Delhi, India.; c Manyata Steering Committee, Federation of Obstetric and Gynaecological Societies of India, Mumbai, India.; d Ariadne Labs, a joint center of the Harvard T.H. Chan School of Public Health and Brigham and Women’s Hospital; Department of Medicine, Harvard Medical School; Division of Global Health Equity, Brigham and Women’s Hospital, Boston, MA, USA.

## Abstract

**Background:**

In India, more than 60% of hospital beds are in private facilities, yet several studies have observed suboptimal quality of care in private facilities. We aimed to understand the role of Manyata, a quality improvement initiative in private facilities focused on mentorship and clinical standards, to improve the knowledge and skills of health care providers, their adherence to key childbirth-related clinical practices, and health outcomes for women and newborns.

**Methods:**

We conducted a secondary analysis of Manyata program data collected from 466 private facilities across 3 states (Jharkhand, Maharashtra, and Uttar Pradesh) in India from October 2016 to February 2019. We calculated means and 95% confidence intervals for knowledge and skills assessment, adherence to facility standards was analyzed by calculating the proportion of facilities passing a given quality standard at baseline and endline, and changes in pregnancy outcomes were assessed with autoregression modeling.

**Results:**

From assessments conducted before and after training among providers in Manyata, we observed a significant increase in average knowledge score (6.3 vs. 13.2 of 20) and skill score (8.0 vs. 34.3 of 40). Overall, a significant increase occurred in adherence to clinical standards between baseline and endline assessments (29% vs. 93%). The standards with the greatest improvements were identification and management of eclampsia/preeclampsia, postpartum hemorrhage, and neonatal resuscitation. There were no significant changes over time in absolute rate of reported complications; however, referral rates from private facilities for preeclampsia and newborn sepsis identification and management declined.

**Conclusion:**

Our analysis indicates private facilities’ adherence to quality standards and nurses’ childbirth knowledge and practical skills increased during Manyata. Additional efforts are needed to ensure high-quality care during cesarean deliveries at private facilities. Future studies with rigorous design are required to evaluate the impact of this quality improvement initiative in improving pregnancy outcomes.

## Abstract (precis)

Manyata, a quality improvement initiative for labor room staff at private facilities in India, yielded increased knowledge among staff, increased adherence to evidence-based practices, and a suggestive decrease in referrals but no change in facility-reported health outcomes.

## Teaser

**Key Findings:**

- After the Manyata quality improvement initiative in private facilities was implemented, nurses’ knowledge and practical skills in maternal and newborn practices increased.
- Private sector facilities’ adherence to quality standards and evidence-based practices improved during their participation in Manyata; however, no change in in-facility morbidity or mortality was reported.
- Certification strategies offered by professional societies, such as the Federation of Obstetric and Gynaecological Societies of India, can be effective in improving quality at private health facilities, as long as training and mentoring support are provided.

**Key Implications:**

- Manyata offers an effective model for improving the quality of care at private maternity facilities in India.
- More work is needed to pursue strategies to ensure that improvements in quality are sustained.

## INTRODUCTION

The United Nation’s Sustainable Development Goals highlight the importance of partnership for achieving health-related goals and universal health coverage.^1^ Attaining the Sustainable Development Goals through public sector engagement alone will not result in desired impacts.^2^ In recent years, the role of the private health care sector in low- and middle-income countries dramatically increased.^3^ Although clients perceive the quality of care in private health facilities to be better due to shorter waiting times, greater hospitality, longer and flexible opening hours, and greater availability of staff, the competence of providers has been found to be suboptimal in both private and public sectors globally.^4^ Therefore, to accelerate progress in achieving the Sustainable Development Goals, it is imperative to focus on improving quality of care both in public and private sectors.^5^

In India, the private sector accounts for up to 80% of all outpatient care and up to 60% of inpatient care.^6^ Moreover, approximately 60% of hospital beds in India are in the private sector, as are the majority of human resources, including 70% of the total health workforce, 80% of physicians, and most obstetricians.^7^ According to the National Family Health Survey 4 (NFHS-4), up to 22% of institutional deliveries in rural areas and up to 43% of institutional deliveries in urban areas occur in private facilities.^8^ Several studies conducted across India report the quality of maternity care in private facilities as suboptimal.^9^^–^^12^ The main contributors to poor quality were lack of qualified staff, technical resources, regulatory guidelines, and quality improvement initiatives.^11^^,^^13^^,^^14^ Moreover, it was observed that nursing staff providing maternity care at these private health care facilities were underqualified, and did not have the skills for evidence-based practices; some of the facilities did not have a single nurse.^9^^,^^11^^,^^12^ Respectful maternity care practices in the private sector, while frequently reported to be better than in public facilities, have room for improvement.^15^ The Government of India launched various initiatives for improving the quality of maternity care in public facilities,^16^^–^^19^ some of which have now shown to have a meaningful impact on reducing neonatal deaths and stillbirths,^20^^,^^21^ yet there are no comparable quality improvement initiatives for private health facilities.

Given the role of private maternity care in India and global evidence on the impact of effective and quality care around childbirth on maternal and neonatal outcomes,^22^^,^^23^ it is crucial to address the quality of care gap in the private sector. Effective strategies are needed to improve the quality of childbirth care in private sector facilities.

Given the role of private maternity care in India, it is crucial to address the quality of care gap in the private sector.

### Program Description

Manyata (Hindi for “accreditation” or “recognition”) is a quality improvement and certification initiative offered by the Federation of Obstetric and Gynaecological Societies of India (FOGSI). The certification acts as a stamp of quality ensuring consistent, safe, and respectful maternity care for women during the antenatal, intrapartum, and postpartum periods. Manyata is based on the World Health Organization’s Safe Childbirth Checklist and adapted under the guidance of FOGSI, with technical assistance from Jhpiego (an international nonprofit health organization affiliated with Johns Hopkins University). The program is centered on 16 quality standards that stakeholders found to be the most important and that were considered achievable at small private health facilities, including antenatal care, prevention of postpartum hemorrhage, adherence to infection and complications protocols, cesarean deliveries, and respectful maternity care (Table 1 includes Manyata standards and Supplement 1 shows detailed verification criteria).

**TABLE 1.** Manyata Clinical Standards for Maternity Care in India

**Standard No.**	**Manyata Clinical Standards**
Antenatal care	
1	Provider screens for key clinical conditions that may lead to complications during pregnancy (to be verified only among booked cases).
At admission	
2	Provider prepares for safe care during delivery (to be checked every day).
3	Provider assesses all pregnant women at admission.
4	Provider conducts pelvic examination appropriately.
5	Provider monitors the progress of labor appropriately.
6	Provider ensures respectful and supportive care.
At delivery	
7	Provider assists the pregnant woman to have a safe and clean birth.
8	Provider conducts a rapid initial assessment and performs immediate newborn care (if baby cried immediately).
9	Provider performs active management of third stage of labor.
10	Provider identifies and manages postpartum hemorrhage.
11	Provider identifies and manages severe preeclampsia/eclampsia.
12	Provider performs newborn resuscitation if baby does not cry immediately after birth.
13	Provider ensures care of newborn with small size at birth.
Beyond delivery	
14	Facility adheres to universal infection prevention protocols.
15	Provider ensures adequate postpartum care package is offered to the mother and baby at discharge.
Cesarean standard	
16	Provider reviews clinical practices related to cesarean delivery at regular intervals.

Facilities are sensitized to Manyata and encouraged to enroll by local and national FOGSI professional societies. The connection to the professional society facilitates participation, accountability, and commitment to the program, although additional qualitative work is underway to explore facilities’ reasons for joining. The participation of health facilities in this initiative is voluntary. To participate in Manyata, a facility has to (1) be registered with local health authorities; (2) provide maternity services; (3) have a facility owner, medical officer, or manager who is a member of FOGSI; and (4) express willingness to participate in the Manyata program by paying a nominal fee and submitting a letter of intent to FOGSI.^9^

**FIGURE 1:**
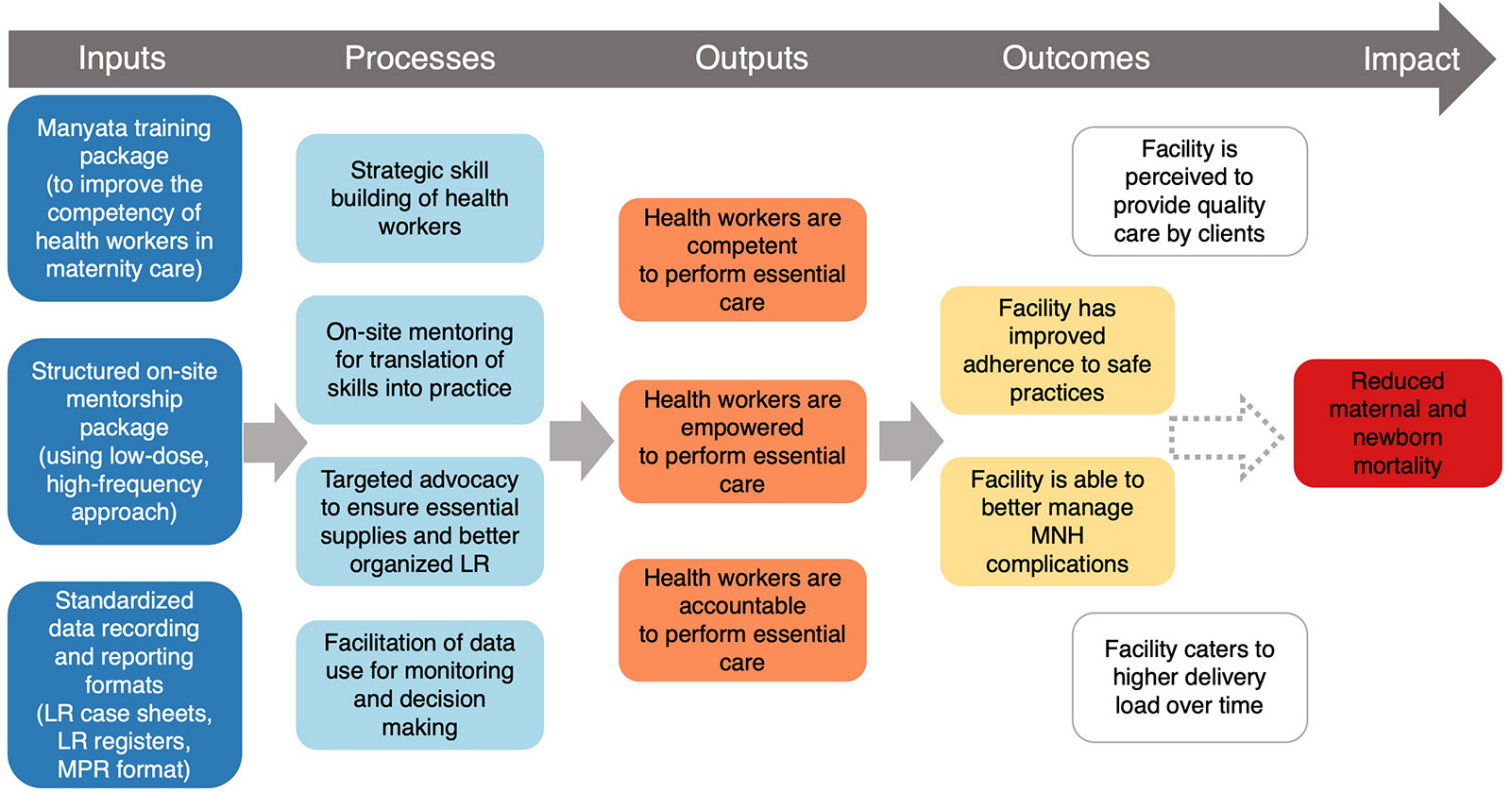
Manyata Theory of Change Abbreviations: LR, labor room; MNH, maternal and neonatal health; MPR, monthly progress report.

Upon initiation of Manyata at a facility, a gap assessment is conducted to identify areas for improvement, and all staff who provide childbirth services (primarily nurses) are eligible to participate in 2 to 3 days of didactic and hands-on mannequin-based training on the Manyata quality standards. The training is led by program officers from Jhpiego with a medical background (Bachelor of Medicine and Bachelor of Surgery or Bachelor of Science in Nursing), and when possible, are additionally supported by FOGSI-affiliated physicians. While not all trainers received Government of India Safe Birth Attendance training, all received technical training on childbirth practices and coaching techniques. Following the training, Jhpiego program officers conduct 5–10 in-person mentoring visits at each facility based on a low-dose high-frequency approach to provide customized support for the translation of skills to practice, and to support problem solving to improve adherence to the 16 Manyata standards. The initial gap assessment at each facility is used to inform the mentorship visits. The average time from enrollment to completion of the program is 5 months, although facilities dictate when these support visits will occur and can opt for a shorter or longer time frame depending on their availability and interest. After completion of training and mentoring visits, facilities are encouraged to apply for Manyata certification. The certification visits are conducted by FOGSI members who are unaffiliated with the facility and who are trained peers (obstetricians) of those who are working in the facility; thus, they are acutely aware of the challenges that private facilities face. Figure 1 shows the Manyata Theory of Change.

Facilities that have full adherence to at least 85% of Manyata’s clinical standards (14 of 16 standards) earn Manyata certification.^24^ To “pass” an individual standard, a facility must pass each of the associated verification criteria (Supplement 1). If a facility achieved more than 50% of the standards but failed to meet the required cutoff for certification, the facility could have an additional month to address identified gaps before a second assessment was conducted, contributing to the program’s high pass rates. If a facility achieved less than 50% of the standards, the facility was asked to continue work on improving adherence and reapply 3 months later.

Several notable challenges were encountered during Manyata. Although each Manyata facility had at least 1 qualified obstetrician, the overall shortage of nurses in India and particularly of skilled and qualified nurses with a recognized degree from an institution—either auxiliary nurse midwife (18-month course) or Bachelor of Science in Nursing (3-year course)—is a well-documented challenge in both the private and public sector.^25^ Thus staff shortages and turnover were a challenge for facilities generally and for program implementation. In particular, the variable skill level of nurses, and at times extremely low levels of training, necessitated additional support and drills to ensure full understanding of the Manyata standards and to build the teamwork needed to improve care delivery. Ensuring training and mentorship coverage for staff working various shifts was another challenge, which was overcome by scheduling trainings close to the change of shifts.

After initial piloting, Manyata was officially launched in 2016 in 3 states at first—Jharkhand, Maharashtra, and Uttar Pradesh. The program has now expanded to additional states/territories (Assam, Delhi, Haryana, Karnataka, Punjab, Rajasthan, and Tamil Nadu) albeit with differing quality improvement models. The activities described in this article were supported by funding from MSD, through its MSD for Mothers program. MSD for Mothers is an initiative of Merck & Co., Inc., Kenilworth, NJ, USA. FOGSI’s leadership and network of local societies continues to support the program in spreading awareness and encouraging involvement among its members.

This study aims to understand the role of Manyata to improve the knowledge and skills of health care providers in private facilities, adherence to key clinical practices, and delivery outcomes for women and their newborns. In this analysis, we describe changes over the course of Manyata in (1) nurses’ knowledge; (2) adherence to 16 key clinical standards and associated evidence-based practices; and (3) in-hospital morbidity, mortality, and referral patterns.

This study aims to understand the role of Manyata to improve the knowledge and skills of health care providers, adherence to key clinical practices, and delivery outcomes for women and their newborns.

## METHODS

### Program Setting

**FIGURE 2:**
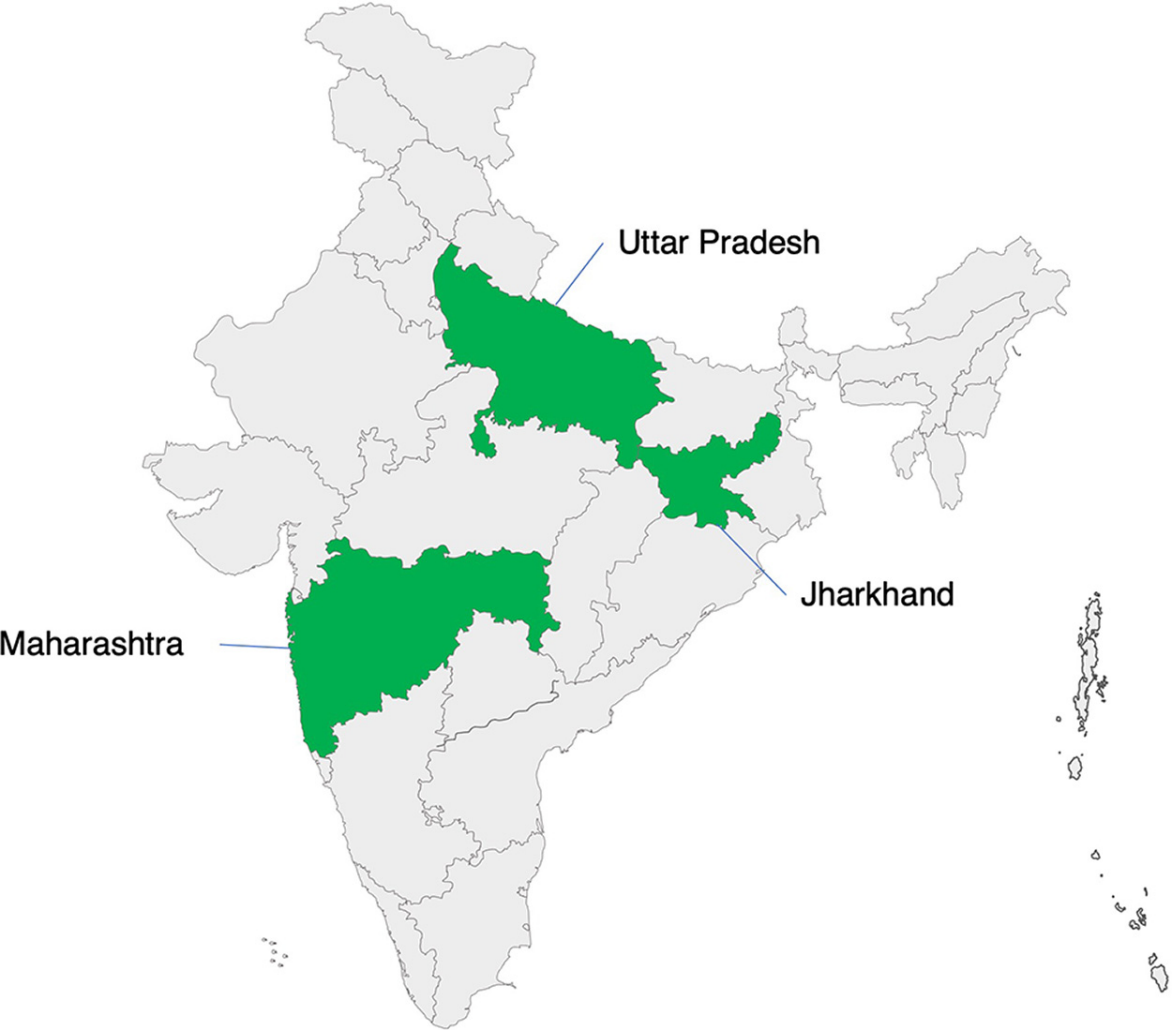
Manyata Project States in India

We conducted a secondary analysis of data collected during Manyata from October 2016 to February 2019. The program was implemented in 3 states in Jharkhand, Maharashtra, and Uttar Pradesh (Figure 2), which were selected based on convenience of where the program was administratively chosen to be implemented.

Uttar Pradesh and Maharashtra are the first and second most populous states in India, respectively, while Jharkhand is a state with a predominantly tribal population.^26^ The estimated maternal mortality ratio per 100,000 live births is 71, 46, and 197 for Jharkhand, Maharashtra, and Uttar Pradesh, respectively.^27^ The percentage of deliveries that occur in private institutions in urban areas varies from state to state (44% in Jharkhand, 55% in Maharashtra, and 43% in Uttar Pradesh).^28^ In the rural areas, childbirth in the private sector accounts for a lower percentage of childbirths (12% for Jharkhand, 35% for Maharashtra, and 21% for Uttar Pradesh).^28^

### Administrative Data Collection

All data were collected as part of the implementation of Manyata and did not take place in a research setting.

#### Knowledge and Competency Assessments

To assess competency of key clinical skills, knowledge assessments and objective structured clinical examinations (OSCEs) were conducted before and immediately following a 2–3 day in-person training at Manyata initiation. In practice, nurses were the primary participants for the training; only nurses’ responses were included in knowledge assessment and OSCE scores. Pre- and post-test scores for the knowledge assessment and OSCEs were not collected in 2017; thus, trainings conducted in 2017 were excluded from this analysis. The knowledge assessment included 20 multiple-choice questions on various evidence-based practices of maternity care, including partograph monitoring, administration of oxytocin, identification and management of preeclampsia, immediate newborn care, administration of antenatal corticosteroids, newborn resuscitation, breastfeeding, discharge counseling, management of low birth weight babies, infection prevention and control, and biomedical waste management. Due to scheduling and clinical duties, not every nurse attended every part of the training nor necessarily completed both the pre- and post-tests. Facility-level scores (not individual) were thus considered to inform a facility’s mentoring plan.

#### Adherence to Manyata Quality Standards

Upon initiation of the program, Jhpiego program officers performed an in-person assessment of each facility to understand baseline adherence to standards. The assessment consisted of a standardized checklist (Table 1 and Supplement 1) that was completed at the facility level in collaboration with facility staff. The 16 quality standards included topics related to respectful maternity care, management of common complications such as eclampsia and postpartum hemorrhage, labor monitoring, universal precautions for infection prevention and control, and care of small newborns, among others. The assessment adapted the methodology and tools approved by the Government of India for quality improvement initiatives in public settings,^29^ which have been used in other studies.^21^^,^^30^ The assessment took an estimated 4 hours to complete for each facility and included a combination of direct observation (preferred), medical record review, and interviews with facility leaders, staff, and patients.

- If a practice was directly observed, then no further verification was needed.
- For medical record review, at least 50% of reviewed records must indicate adherence.
- For leader and staff interviews, 100% of interviews must indicate adherence.
- For patient interviews, at least 50% of patients must indicate adherence.

Given the programmatic nature of the data, there was no predetermined sample size. Assessors used a cross-sectional sample of medical records and did not follow individual women over time or across trimesters. Once the facility completed its mentorship visits and quality improvement journey, the facility could request a final endline assessment to determine if the standards for certification were met. This endline assessment was completed by trained FOGSI assessors, using the same Manyata quality standards checklist that was used at baseline. All assessors received standardized training on assessment practices.

#### Health Outcomes and Complication Management

From program initiation and throughout Manyata participation, each facility was encouraged to submit monthly progress reports on select indicators, including adherence to Manyata quality standards, morbidities (postpartum hemorrhage, eclampsia, preeclampsia, obstructed labor, neonatal sepsis, neonatal asphyxia, and prematurity), mortality (intrauterine death, any neonatal death, and maternal death), and process measures such as referrals and cesarean deliveries. The monthly progress reports included counts of the total number of births in that month and the number of cases of each outcome. These reports were not required but were meant to help facilities track their progress over time. Monthly progress report submission could continue even after certification was completed.

### Data Analysis

For the knowledge assessments and OSCE scores, we calculated means and 95% confidence intervals (CIs) for pre- and post-test scores. We also calculated scores as percentages using the mean number of questions correct/total number of questions (max score). “Passing” scores for each of the OSCEs were prespecified as achieving at least 75% or more of the required skills; there was no predetermined passing score for the knowledge test.

To understand facility-level adherence to quality standards, we calculated the percentage of facilities that met each standard at baseline and endline. To understand changes in complication management, in-facility morbidity, and in-facility mortality over time, we used monthly reporting data from facilities that reported at least 3 months of data between their baseline and endline assessments. For modeling purposes, we excluded facilities that were missing either at baseline or endline assessment, as well as any facility that reported zero complications and mortality over the course of the program.

As shown in Table 2, we modeled the absolute rates of patient-level events over time (N outcomes/N births) for all outcomes. We also modeled referral rates over time (N referrals/N outcomes), such as the number of referrals for obstructed labor in a month divided by the total number of cases of obstructed labor in that month. Referrals that occurred after a cesarean delivery were not recorded.

**TABLE 2.** Maternal Outcomes for Modeling in Manyata Facilities in Jharkhand, Maharashtra, and Uttar Pradesh States, India

	**Modeling Change Over Time (Patient-Level)**	
**Outcome**	**In Absolute Rates:** **N Events/** **N Births**	**In Referral Rates:** **N Referrals/** **N Events**
Morbidities		
Hemorrhage	Y	Y
Eclampsia or preeclampsia (pooled)	Y	Y
Obstructed labor	Y	Y
Asphyxia	Y	Y
Maternal sepsis	Y	Y
Newborn sepsis	Y	Y
Prematurity	Y	Y
Mortality		
Intrauterine death	Y	N/A
Any neonatal death	Y	N/A
Process measure		
Cesarean delivery	Y	Not recorded

Abbreviation: N/A, not applicable.

We built repeated measures models of the form:

$$\frac{N_{\text{events}}}{N_{\text{trials}}}=\beta_{0}+\beta_{1}\;\text{Month}\;+\;\beta_{z}Z+\varepsilon$$

where N_events_ is the number of events in a month (numerator); N_trials_ is the number of events in a month (denominator); Month is the number of months since the start of the Manyata program (continuous integer); Z is the vector of 4 facility characteristics: state (nominal), number of beds, staff, and birthload (continuous); β_x_ is the coefficient estimate; and ε is the error term.

We used an autoregressive correlation structure to account for the repeated measurements over facilities.

To account for differences in the length of time facilities participated in Manyata, we weighted the data in these models by the number of months between baseline and endline. To obtain the weights, we built a Poisson regression model of the form:

$$N_{\text{Months}}=\beta_{0}+\;\beta_{z}Z+\varepsilon$$

where all elements are as above and N_months_ is the count of months between a facility’s baseline and endline assessments.

We estimated the weight for each facility as the inverse of the predicted value from this model. All analyses were performed on data using SAS software version 9.4.

### Ethical Approval

All data used in this study were collected as a part of routine monitoring of the program. All facilities that enrolled in Manyata agreed to take part in monitoring and evaluation activities related to the program. Data were shared back with individual facilities for the purposes of improvement. The Johns Hopkins School of Public Health Institutional Review Board (IRB) deemed the Manyata program activities and routine program data collection to be nonhuman subjects research, thus not requiring IRB oversight (IRB No: 00009525). The data set used for this analysis was deidentified. The Harvard T.H. Chan School of Public Health reviewed the protocol for secondary data analysis and determined that it was also nonhuman subjects research.

## RESULTS

**FIGURE 3:**
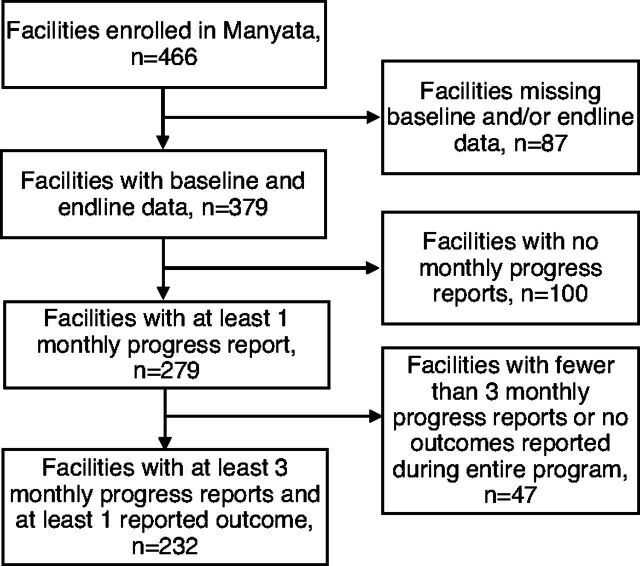
Flow Chart of Manyata-Enrolled Facilities in Jharkhand, Maharashtra, and Uttar Pradesh States, India

From 2016 to 2019, 466 private facilities enrolled in the Manyata program (Table 3) across 3 states in India (Jharkhand n=103, 22%; Maharashtra n=134, 29%; Uttar Pradesh n=229, 49%). The majority of these facilities had fewer than 30 staff (n=332, 71%) and had less than an average of 30 or fewer births per month (n=273, 59%). Final Manyata certification was achieved by 377 facilities (81%). Among the 89 facilities (19%) that did not receive certification, almost all (n=83) did not complete the program and never attempted to gain certification. Only 4 facilities tried to receive certification but failed; 2 additional facilities requested assessment but were never assessed. Figure 3 describes the population included in each analysis.

**TABLE 3.** Characteristics of Manyata Facilities in Jharkhand, Maharashtra, and Uttar Pradesh States, India

**Characteristics**	**Facilities, No. (%)** **(N=466)**
State	
Jharkhand	103 (22.1)
Maharashtra	134 (28.8)
Uttar Pradesh	229 (49.1)
Size of facility, beds^a^	
≤20	208 (44.6)
>20	206 (44.2)
Missing	52 (11.2)
Number of staff^a^	
≤15	172 (36.9)
16–30	160 (34.3)
>30	82 (17.6)
Missing	52 (11.2)
Time between baseline and endline assessments, months^a^	
≤3	83 (17.8)
4–6	174 (37.3)
7–9	90 (19.3)
≥10	32 (6.9)
Missing	87 (18.7)
Type of facility^a^	
Exclusive maternity hospital	186 (39.9)
Multispecialty hospital	202 (43.4)
Others	1 (0.2)
Missing	77 (16.5)
Average monthly delivery load^a^	
1–10	70 (15.0)
11–20	117 (25.1)
21–30	86 (18.5)
≥31	112 (24.0)
Missing	81 (17.4)

^a^ These characteristics have more than 10% missing data, and thus should be interpreted with caution.

### Knowledge and Skills Assessments

Baseline knowledge scores related to childbirth and management of complications were low; average scores on the knowledge test varied with 16% in Jharkhand, 36% in Maharashtra, and 31% in Uttar Pradesh (overall shown in Table 4; state-wise shown in Supplement 2). After the Manyata training, knowledge scores improved dramatically in Uttar Pradesh (final score=83%) but remained low in Jharkhand (final score=56%) and Maharashtra (final score=56%). OSCEs were completed related to the active management of the third stage of labor (AMTSL), newborn resuscitation, antenatal complications, and postnatal complications. Average baseline OSCE scores ranged from 8.8% to 31.7% (Table 4 and Supplement 2). In the OSCE post-test, average passing scores were achieved in AMTSL (91.0%) and antenatal complications (76.3%) but not in newborn resuscitation (65.6%) or management of postnatal complications (70.8%). Knowledge and OSCE scores were similar in small (1 to 20 beds), medium (21 to 50 beds) and large facilities (51+ beds) (Supplement 3).

After the Manyata training, nurses’ knowledge scores improved in Uttar Pradesh but remained low in Jharkhand and Maharashtra.

**TABLE 4.** Knowledge and Skills Assessments for Nurses Participating in Manyata in Jharkhand, Maharashtra, and Uttar Pradesh States, India

	**Total Nurses, No.**	**Average Prescore/ Max Score (%)**	**95% CI**	**Average Postscore/Max Score (%)**	**95% CI**
Knowledge assessment					
Knowledge test scores^a^	912	6.3/20 (31.5)	6.1, 6.5	13.2/20 (66.0)	12.9, 13.4
OSCE assessment					
Overall^a^	878	8.0/46 (17.4)	7.6, 8.3	34.3/46 (74.6)	34.1, 34.6
AMSTL (passed=8 or higher)	888	1.5/10 (15.0)	1.4, 1.7	9.1/10 (91.0^b^)	9.1, 9.2
Antenatal complications (passed=6 or higher)	882	1.3/8 (16.3)	1.2, 1.4	6.1/8 (76.3^b^)	6.0, 6.3
Newborn resuscitation (passed=12 or higher)	890	1.4/16 (8.8)	1.3, 1.5	10.5/16 (65.6^c^)	10.4, 10.6
Postnatal complications (passed=9 or higher)	880	3.8/12 (31.7)	3.6, 4.0	8.5/12 (70.8^c^)	8.4, 8.7

Abbreviations: AMSTL, active management of third stage of labor; CI, confidence interval; OSCE, objective structured clinical examinations.

^a^ Passing score not specified.

^b^ Average postscore was above the “passing” level (>75%).

^c^Average postscore was below the “passing” level (<75%).

### Behavior Change/Adherence to Quality Standards

**FIGURE 4:**
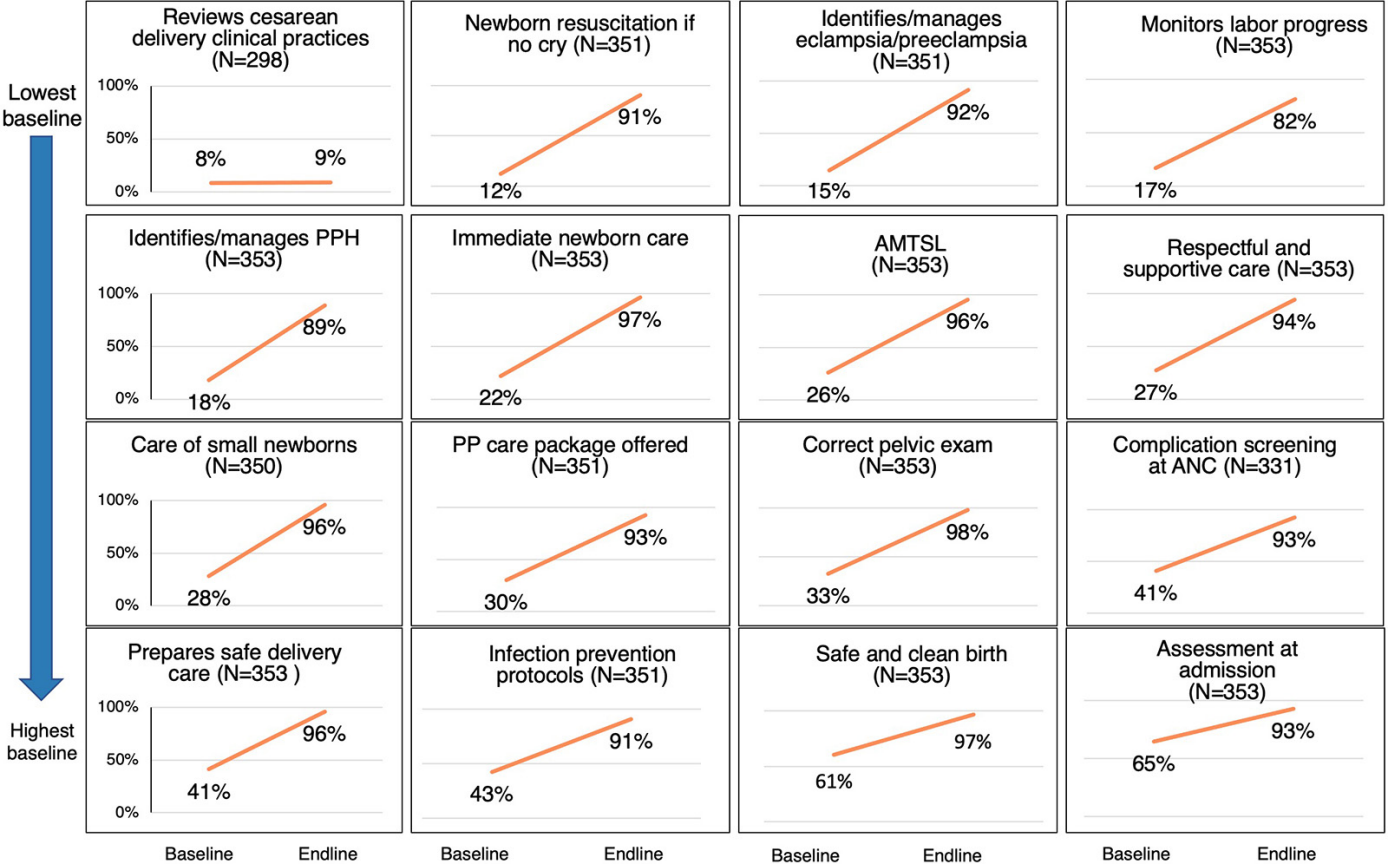
Facilities’ Adherence to Manyata Quality Standards at Baseline and Endline in Jharkhand, Maharashtra, and Uttar Pradesh States, India Abbreviations: AMTSL, active management of third stage of labor; ANC, antenatal care; PP, postpartum; PPH, postpartum hemorrhage.

Among the 379 facilities with both baseline and endline assessments, facilities on average adhered to 29% of standards at the start of the program. Baseline adherence varied by geographic location and size of facility (percentage of standards passed at baseline by state: Jharkhand, 37.0%; Maharashtra, 18.7%; and Uttar Pradesh, 33.0%. Percentage of standards passed at baseline by size of facility: 20 beds or less, 25.3% and more than 20 beds, 33.8%). Standards that were most likely to be missed during the baseline assessment included those related to cesarean delivery (achieved by 8% of facilities), newborn resuscitation (achieved by 12% of facilities), and management of eclampsia/preeclampsia (achieved by 15% of facilities) (Figure 4). At baseline, standards related to respectful maternity care were met by 27% of facilities.

By the endline assessment, facilities on average adhered to 93% of standards (percentage of standards passed at endline by state: Jharkhand: 92.8%, Maharashtra: 92.3%, Uttar Pradesh: 93.5%; percentage of standards passed at baseline by size of facility: 20 beds or less: 92.3%; more than 20 beds: 93.6%). The standards that showed the greatest improvements with Manyata included those on the management of eclampsia/preeclampsia and on neonatal resuscitation (Figure 4). The standard that was the most likely to be missed during the endline assessment was related to cesarean delivery (achieved by 9% of facilities). The next lowest scores were notably higher: labor monitoring (achieved by 82% of facilities) and the identification and management of postpartum hemorrhage (achieved by 89% of facilities). At the endline assessment, 94% of facilities met the respectful maternity care standards.

The standards that showed the greatest improvements with Manyata included those on the management of eclampsia/preeclampsia and on neonatal resuscitation.

Length of participation in the program, as measured by length of time between baseline assessment and endline assessment, varied considerably across facilities. The median time between program enrollment and certification was 5 months (range=0–23 months). There was no difference in the median time from enrollment to certification across geographies (Jharkhand, Maharashtra, Uttar Pradesh). A higher percentage of larger facilities (more than 20 beds, more than 30 staff), facilities with higher delivery loads (30+ births per month), and facilities in Jharkhand were more likely to sustain the program as evidenced by their continued submission of monitoring data postcertification.

### Health Impact

**FIGURE 5:**
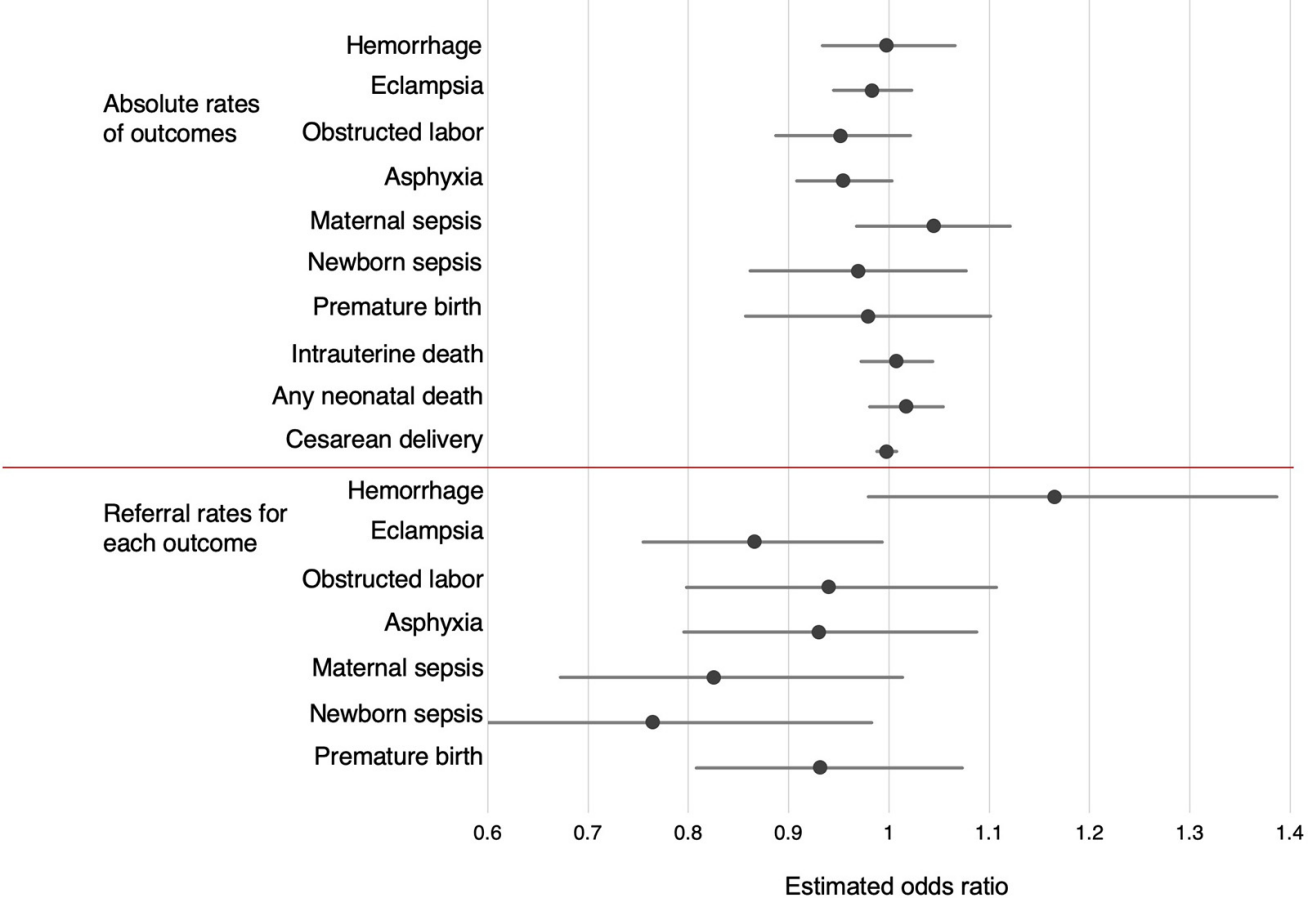
Estimated Change in Specific Health Outcomes With Each Month of Manyata in Jharkhand, Maharashtra, and Uttar Pradesh States, India

A total of 279 facilities submitted at least 1 monthly report; among those facilities, 232 reported at least 1 complication or mortality over the course of the program, and self-reported at least 3 months’ worth of data on morbidity, mortality, and complication management (range: 3–25 monthly reports submitted per facility). Our models found no significant change over time in the absolute rate of any outcomes (Figure 5). Given the major differences in morbidity and mortality rates across the 3 states (Jharkhand, Maharashtra, and Uttar Pradesh), we assessed changes in these complications over time for each state individually but found no significant change in these health measures (data not shown).

Regarding referral rates, 2 models suggested declining referral rates over time for patients with preeclampsia or eclampsia (odds ratio [OR]=0.87; 95% CI=0.75, 0.99), and for newborns with sepsis (OR=0.76; 95% CI=0.60, 0.98) (Figure 5; Table 5).

**TABLE 5.** Monthly Changes in Referral Practices for Specific Complications During Manyata in Jharkhand, Maharashtra, and Uttar Pradesh States, India

**Outcome**	**Parameter Estimate**	***P* Value**	**Odds Ratio**	**95%** **Confidence Interval **
Hemorrhage	0.15	.08	1.17	0.98, 1.39
Eclampsia or preeclampsia (pooled)	−0.14	.04	0.87	0.75, 0.99
Obstructed labor	−0.06	.46	0.94	0.80, 1.11
Asphyxia	−0.07	.37	0.93	0.80, 1.09
Maternal sepsis	−0.19	.07	0.83	0.67, 1.01
Newborn sepsis	−0.27	.04	0.76	0.60, 0.98
Premature	−0.07	.32	0.93	0.81, 1.07

## DISCUSSION

### Summary of Findings

Our results show that over the Manyata program period, nurses’ childbirth knowledge and practical skills increased, facilities’ adherence to quality standards rose, referral patterns of select complications slightly reduced, but in-facility morbidity and mortality did not change. The 466 private sector facilities that elected to join Manyata were diverse and included both large hospitals with 100 or more beds (n=20) and very small facilities with 10 or fewer beds (n=89), and were situated in vastly different environments, both socioeconomically and in terms of the burden of disease. For example, the maternal mortality ratio in Maharashtra is 46 deaths per 100,000 live births compared to 197 deaths per 100,000 live births in Uttar Pradesh.^27^ Accordingly, not all facilities required the same support nor achieved Manyata certification on the same timeline.

Our results show that over the Manyata program period, nurses’ childbirth knowledge and practical skills increased, facilities’ adherence to quality standards rose, referral patterns of select complications slightly reduced.

The training was a success when viewed in relative terms, with average knowledge scores increasing by 110% and OSCE scores showing 124% to 650% increases. Despite these improvements, the final average knowledge scores and OSCE scores for newborn resuscitation and postnatal complications remained below the passing mark of 75%. Given the low starting point for knowledge and OSCE scores, this shortcoming ultimately reinforces the limitations of a 3-day training and the need for the ongoing mentoring aspect of Manyata.^31^

During the mentorship and quality improvement portion of the program, which involved monthly in-person visits for an average of 5 months, facilities’ adherence to quality standards increased dramatically among facilities that completed the program, particularly in the areas of neonatal resuscitation and preeclampsia/eclampsia management. Given the high certification rate among facilities, the mentorship model, which addressed both skill gaps and facility process improvements (organization of labor rooms, ensuring the availability of essential commodities, etc.), appears to create an effective bridge to help facilities build on the initial training and reach a high level of quality within several months. This is consistent with a recent systematic review by Rowe et al. which found that in addition to training, supportive supervision and problem-solving support are needed to improve quality and institutionalize the performance of evidence-based practices.^31^

Given the high certification rate among facilities, the mentorship model appears to create an effective bridge to help facilities build on the initial training and reach a high level of quality within several months.

Improvements in the respectful maternity care standard (standard #6), particularly related to privacy, permission to have a birth companion, confidentiality, and patient communication, improved greatly with Manyata and may have a strong influence on patients’ experience of care during childbirth. In contrast, adherence to the cesarean delivery standard (standard #16) was notably low. Only 298 (of 379) facilities were assessed on that standard and among facilities that were assessed, average adherence was less than 10% on the endline assessment. As cesarean deliveries comprise 60% of births at these facilities, this is an important issue that needs further exploration to understand how to assure quality in this area. Among the facilities that enrolled in Manyata, 19% did not gain certification; the majority of these facilities never requested it and presumably dropped out. Additional studies should explore causes of program termination and mitigation methods to reduce the dropout rate.

We did not see a change in in-facility morbidity or mortality reported by facilities during their participation in Manyata. Since health outcomes were self-reported by facilities and were generally only submitted during the 5 months between enrollment and accreditation, the period of data collection may have been too short to significantly affect morbidity or mortality. Additionally, data collected for this analysis was intended for programmatic monitoring and evaluation and was not initially powered or structured to measure changes in mortality, which limit the conclusions that we are able to draw. The possible decrease in referral practices for preeclampsia/eclampsia and newborn sepsis may imply that the program strengthened the ability of facilities to manage complications in-house, which can potentially relieve the high delivery loads at many large public facilities. However, from our data, it is unclear if this decrease in referrals ultimately resulted in better health outcomes for patients, compared with patients who were referred.

Learnings from similarly structured programs based on adapted versions of the World Health Organization Safe Childbirth Checklist in the public sector may shed some light on potential connections between care practices and mortality. For example, the BetterBirth randomized control trial study in Uttar Pradesh, India,^32^ which included an 8-month intervention and 12-month data collection period, found that differences in mortality were only seen in facilities with very high adherence to at least 85% of the quality standards. This pattern is seen in other studies as well.^33^^,^^34^ In Manyata, since health outcomes were self-reported by the facilities and collected during the approximately 5-month program implementation (and generally stopped once accreditation was reached), it is possible that change in maternal and neonatal health outcomes requires longer duration of exposure and data collection as was found in other similar programs in India’s public sector.^20^^,^^21^^,^^30^

A recent analysis of the Dakshata program,^21^ a similarly structured program for the public sector in India, showed that the program was associated with a decrease in maternal morbidity and stillbirths when evaluated at a large scale (17 million births) over a longer time frame. Moreover, a quasi-experimental observational study of the Safe Childbirth Checklist conducted over a period of 2 years in high delivery load facilities in India revealed the increased adherence to evidence-based practices^20^ that resulted in an 11.16% reduction in early neonatal deaths and an 11.3% reduction in stillbirth rates. A larger analysis of the Manyata program may bear similar results once the program is further established, has wider participation, and outcomes from private facilities can reliably be measured over a longer duration.

#### Contributions to Knowledge/Science

Private hospitals are increasingly recognized as a major part of the health ecosystem around childbirth. In India, approximately 40% of deliveries occur in the private sector,^28^ and this trend is increasingly seen in countries around the globe, including Pakistan (44% of deliveries),^35^ the Democratic Republic of Congo (20.5% of deliveries),^36^ and Indonesia (28% of deliveries).^23^^,^^37^ Despite providing a major source of childbirth care, the private sector health facilities in India and beyond have uneven regulatory oversight and are often excluded from national or state-driven quality improvement initiatives that are largely targeted to improve the public sector.^4^

Evidence-based strategies to improve the quality of care in private-sector childbirth facilities are needed. The Manyata program model provides a realistic example of how existing professional societies can be supported to encourage and monitor quality. In this case, the FOGSI leadership defined the pragmatic, evidence-based standards as the minimum desirable care for ensuring safe delivery. The FOGSI network helped to increase interest and demand for the program and used fellow FOGSI members to administer the quality assurance aspect of the program (i.e., determine if the facility passed 85% of the Manyata quality standards during the final assessment). As government regulations alone often have a limited effect on childbirth quality in the private sector,^38^ our analysis of programmatic data suggests that Manyata could provide a positive, complementary approach to quality that ultimately makes the improvements more sustainable. Recurring oversight and assurance from FOGSI could support the sustainability of this program and may help to create a peer-driven culture of quality among private facilities.

Our analysis of programmatic data suggests that Manyata could provide a positive, complementary approach to quality that ultimately makes improvements in childbirth quality more sustainable.

Certification programs alone are unlikely to drive meaningful, sustained improvements without additional quality improvement support at the facility level. Training and mentoring programs to improve childbirth practices are common throughout the world, yet poor quality data exists on the effectiveness of various strategies.^31^ Group-based, in-service training has shown little effect on changing health care workers’ behavior when used alone. However, the additional strategies of face-to-face mentoring visits and a focus on problem solving, both shown to be more impactful,^31^ as well as continued skills practice and facility process improvement, may have increased Manyata’s effectiveness in increasing knowledge and facility-level adherence to Manyata quality standards.

### Implications and Next Steps

Market forces have not been sufficient to ensure consistent, high-quality childbirth care in the private sector. Programming to support quality improvement and quality assurance is possible and necessary at these facilities, although additional support is needed to address quality gaps related to cesarean deliveries. Strategies to monitor and sustain quality of care postcertification are needed, as currently there are no continued requirements to maintain the Manyata certification. Ongoing efforts are underway to increase the overall sustainability of the Manyata program and decrease reliance on external funding; further study is needed on this topic. Additional research is also needed to further explore health outcomes associated with Manyata.

Strategies to monitor and sustain quality of care postcertification are needed, as currently there are no continued requirements to maintain the Manyata certification.

### Limitations

We share a practical assessment of a quality improvement and assurance program designed to improve private-sector childbirth. Although a randomized control trial would have offered stronger evidence, it was impractical in this setting. The programmatically collected data presented several challenges, including missingness about characteristics of facilities that dropped out of the program. We noted the extent of the missingness in Table 2; missingness is likely due to the self-reported, programmatic nature of the facility characteristics data, and respondents’ potential unease with disclosing facility details due to tax ramifications. The before and after knowledge assessments and OSCE data did not necessarily include the same staff in both the before and after time periods because clinical duties prevented the entire staff from taking both assessments at some facilities. Knowledge and OSCE scores should thus be viewed on a facility level rather than an individual level. The baseline and endline assessments of the Manyata standards were conducted by different data collectors from different organizations, which may have accounted for some of the differences in the scores. However, each data collector underwent the same training and used the same checklist, which attempted to overcome this possible limitation.

The optional, monthly reporting on morbidity and mortality that was self-reported by the facilities themselves likely undercounted morbidities and mortality, due in part to facilities’ reluctance to disclose negative patient information, and because the measures only took into account in-facility outcomes and did not capture any outcomes occurring after discharge. We accounted for the irregular monthly reporting through statistical modeling (as discussed in the methods section). Additionally, we excluded from the modeling any facility that reported zero morbidity and mortality during the reporting period to avoid skewing the results. It is possible that these facilities that reported zero morbidity and mortality during Manyata had higher morbidity and mortality at baseline (before data collection) and that we underestimated the effect of the program on health outcomes.

## CONCLUSION

Manyata involved a combination of quality improvement activities (training and mentoring) led by an international NGO and quality assurance monitoring led by FOGSI. Over the Manyata period, programmatic data suggest an increase in both private facilities’ adherence to quality standards and nurses’ childbirth knowledge and practical skills. Further attention is needed to ensure high-quality care during cesarean deliveries at private facilities. Additional studies are needed to understand the sustainability of high-quality care at private facilities and of the Manyata model overall.

## Supplementary Material

GHSP-D-22-00093-Supplements.pdf

## Notes

**Peer Reviewed**
