# Supplementary material for: Does Quality Certification Work? An Assessment of Manyata, a Childbirth Quality Program in India’s Private Sector
Source: Glob Health Sci Pract. 2022 Dec 21;10(6):e2200093. doi: 10.9745/GHSP-D-22-00093 (PMC9771457; doi:10.9745/GHSP-D-22-00093)
Supplement: GHSP-D-22-00093-Supplements.pdf [file GHSP-D-22-00093-Supplements.pdf]

Supplement to: Delaney MM, Usmanova G, Nair TS, et al. Does quality certification work? An assessment of Manyata, a childbirth quality program in India's private sector. *Glob Health Sci Pract*. 2022;10(6):e2200093. https://doi.org/10.9745/GHSP-D-22-00093

Supplement 1. Manyata Clinical Standards With Detailed Sub-Standards

S.No.	Standard	Verification criteria		Remarks
**Antenatal Care**				
**1**	**Provider screens for key clinical conditions that may lead to complications during pregnancy. (To be verified only among booked cases)**			
1.1	Screens for anemia	1.1.1	Estimates Hb at least once in every trimester	
1.2	Screens for hypertensive disorders of pregnancy	1.2.1	Functional BP instrument and stethoscope at point of use is available	
		1.2.2	Records BP at each ANC visit	
		1.2.3	Performs proteinuria testing during all ANC contacts if a pregnant woman is hypertensive	
1.3	Screens for DM	1.3.1	Uses/Refers for standard single step 75gm OGTT for screening of GDM at first ANC visit and repeats OGTT test at second ANC visit (24 -28 weeks) if negative in first screening	
1.4	Screens for HIV	1.4.1	Screens/ refer for HIV during first ANC visit in all cases, and repeat HIV testing, considering window period if the spouse is positive or s/he have high-risk behavior*	
1.5	Screens for syphilis	1.5.1	Screens/ refer for syphilis in first ANC visit in all cases, and again in the third trimester or at the time of delivery if she has high-risk behavior** or untested earlier.	
1.6	Screens for malaria	1.6.1	Screens for malaria (only in endemic areas)	
1.7	Establishes blood group and Rh type during first ANC visit	1.7.1	Establishes blood group and Rh type during first ANC visit	
1.8	Screens for asymptomatic bacteriuria	1.8.1	Screens for asymptomatic bacteriuria using urine culture/urine gram staining/dipstick test for nitrite during each scheduled ANC contact	
**Standard 1 final response (Yes/No):**				

**At Admission:**				
**2**	**Provider prepares for safe care during delivery (to be checked every day)**			
2.1	Ensures sterile/ HLD delivery tray is available	2.1.1	Ensure availability of Uterotonics agents - IM/IV oxytocin (preferred), misoprostol, PPH Box, Eclampsia kits are ready	
2.2	Ensures functional items for newborn care and resuscitation	2.2.1	Designated new born corner is present	
		2.2.2	Ensures functional items for newborn care and resuscitation	
		2.2.3	Switches radiant warmer 'on' 30 min. before childbirth	
**Standard 2 final response (Yes/No):**				
**3**	**Provider assesses all pregnant women at admission**			
3.1	Takes obstetric, medical and surgical history	3.1.1	Takes obstetric, medical and surgical history	
3.2	Assesses gestational age correctly	3.2.1	Assesses gestational age through either LMP or Fundal height or USG (previous or present is available)	
3.3	Records fetal heart rate	3.3.1	Functional Doppler/ fetoscope/ stethoscope at point of use is available	
		3.3.2	Records FHR	
3.4	Records mother's BP and temperature	3.4.1	Functional BP instrument and stethoscope and functional thermometer at point of use is available	
		3.4.2	Records BP and temperature. Conducts abdominal examination ensuring privacy	
**Standard 3 final response (Yes/No):**				

Supplement to: Delaney MM, Usmanova G, Nair TS, et al. Does quality certification work? An assessment of Manyata, a childbirth quality program in India's private sector. *Glob Health Sci Pract*. 2022;10(6):e2200093. https://doi.org/10.9745/GHSP-D-22-00093

**4**	**Providers conducts Pelvic Exam (PV) examination appropriately**			
4.1	Conducts PV examination as per indication	4.1.1	Conducts PV examination only as indicated (4 hourly or based on clinical indication)***(Ask Doctor/ Nurse as per facility protocol)***	
4.2	Conducts PV examination following infection prevention practices and records findings	4.2.1	Soap, running water, antiseptic solution, sterile gauze/pad is available	
		4.2.2	Performs hand hygiene (washes hands and wears sterile gloves on both the hands with correct technique)	
		4.2.3	Cleans the perineum appropriately before conducting PV examination	
		4.2.4	Alert specialist/doctor if liquor is meconium stained	
		4.2.5	Records findings of PV examination	
**Standard 4 final response (Yes/No):**				
**5**	**Provider monitors the progress of labor appropriately**			
5.1	Undertakes timely assessment of cervical dilatation and descent to monitor the progress of labor	5.1.1	Partograph are available in labor room	
		5.1.2	Initiates partograph plotting when cervical dilatation is >=4 cms.	
5.2	Interprets partograph (condition of mother and fetus and progress of labor) correctly and adjusts care according to findings	5.2.1	If parameters are not normal, identifies complications, records the diagnosis and makes appropriate adjustments in the birth plan ***(Ask Doctor/ Nurse as per facility protocol)***	
5.3	Obstructed labour	5.3.1	Staff knows Diagnosis & Management of Obstructed Labour (Interpreting partograph, Re-hydrates the patient, check vitals, gives broad spectrum antibiotics, perform bladder catheterization and takes blood for Hb & grouping)	

5.4	Unnecessary augmentation and induction of labour is not done using uterotonics.	5.4.1	Oxytocin and misoprostol inductions done only for clear medical indication and the expected benefits outweigh the potential harms. Outpatient induction of labour is not done	
**Standard 5 final response (Yes/No):**				
**6**	**Provider ensures respectful and supportive care**			
6.1	Encourages and welcomes the presence of a birth companion during labor	6.1.1	Encourages and welcomes the presence of a birth companion during labor	
6.2	Treats pregnant woman and her companion cordially and respectfully (RMC), ensures privacy and confidentiality for pregnant woman during her stay. Behavior of labour room staff is dignified and respectful.	6.2.1	There are provisions for privacy in LR (curtains /partition between tables and non-see through windows	
		6.2.2	Treats pregnant woman and her companion cordially and respectfully. Confidentiality of patient's records and clinical information is maintained.	
6.3	Explains danger signs and important care activities to pregnant woman and her companion	6.3.1	Explains danger signs and important care activities to mother and her companion	
**Standard 6 final response (Yes/No):**				
**At Delivery:**				
**7**	**Provider assists the pregnant woman to have a safe and clean birth**			
7.1	Provider ensures six 'cleans' while conducting delivery	7.1.1	Sterile gloves are available	
		7.1.2	Antiseptic solution (Betadine/ Savlon) is available	
		7.1.3	Sterile cord clamp is available	
		7.1.4	Sterile cutting edge (blade/scissors) is available	

7.2	Performs an episiotomy only if indicated with the use of appropriate local anesthetic	7.2.1	Performs an episiotomy only if indicated and uses local anesthesia (***Ask doctor/nurse as per facility protocol)***	
7.3	Allows spontaneous delivery of head by maintaining flexion and giving perineal support; manages cord round the neck; assists in delivery of shoulders and body	7.3.1	Allows spontaneous delivery of head by maintaining flexion and giving perineal support; manages cord round the neck; assists in delivery of shoulders and body	
**Standard 7 final response (Yes/No):**				
**8**	**Provider conducts a rapid initial assessment and performs immediate newborn care (if baby cried immediately)**			
8.1	Delivers the baby on mother's abdomen	8.1.1	Two towels at normal room temperature or pre warmed to room temperature	
		8.1.2	Delivers the baby on mother's abdomen	
8.2	Ensures immediate drying, and asses breathing	8.2.1	If breathing is normal, dries the baby immediately and wraps in second warm towel	
8.3	Performs delayed cord clamping and cutting	8.3.1	Performs delayed cord clamping and cutting (1-3 minutes) unless medical indication otherwise	
8.4	Ensures early initiation of breastfeeding	8.4.1	Initiates breast feeding within one hour of birth	
8.5	Assesses the newborn for any congenital anomalies	8.5.1	Provider immediately assess the newborn for any congenital anomalies	
		8.5.2	Provider ensures specialist care if required	
8.6	Weighs the baby and administers Vitamin K. **OPV/BCG/Hepatitis B vaccinations given within 24 hours of birth**	8.6.1	Baby weighing scale is available	
		8.6.2	Vitamin K injection is available	
		8.6.3	Weighs the baby and administers Vitamin K. **OPV/BCG/Hepatitis B administered within 24 hours of birth**	
**Standard 8 final response (Yes/No):**				

Supplement to: Delaney MM, Usmanova G, Nair TS, et al. Does quality certification work? An assessment of Manyata, a childbirth quality program in India's private sector. *Glob Health Sci Pract*. 2022;10(6):e2200093. https://doi.org/10.9745/GHSP-D-22-00093

**9**	**Provider performs Active Management of Third Stage of Labor (AMTSL)**			
9.1	Performs AMTSL and examines the placenta thoroughly	9.1.1	Palpates mother's abdomen to rule out second baby	
		9.1.2	Administers Uterotonics. Preferred is Inj. Oxytocin 10 I.U. IM/IV within one minute of delivery of baby (use Misoprostol 600 micrograms if oxytocin is not available)	
		9.1.3	***Performs controlled cord traction (CCT) during contraction***	
		9.1.4	***Performs uterine massage***	
		9.1.5	Checks placenta and membranes for completeness before discarding	
**Standard 9 final response (Yes/No):**				
**10**	**Provider identifies and manages Postpartum Hemorrhage (PPH)**			
10.1	Assesses uterine tone and bleeding per vaginum regularly after delivery	10.1.1	Assesses uterine tone and bleeding per vaginum regularly	
10.2	Identifies shock	10.2.1	Identifies shock by signs and symptoms (pulse > 110 per minute, systolic BP < 90 mmHg, cold clammy skin, respiratory rate > 30 per minute, altered sensorium and scanty urine output < 30 ml per hour)	
10.3	Manages shock	10.3.1	Ensures availability of wide bore cannulas (No. 14/16), IV infusion sets and fluids and containers for collection of blood for hemoglobin, blood grouping and cross matching	
		10.3.2	Shouts for help, follows ABC approach, monitors vitals, elevates the foot end and keeps the woman warm	
		10.3.3	Starts IV infusions, collects blood for Hb and grouping and cross matching, catheterizes the bladder and monitors I/O, gives oxygen at the rate of 6-8 liters per minute	
		10.3.4	Identifies specific cause of PPH	

10.4	Manages atonic PPH	10.4.1	Initiates 20 IU oxytocin drip in 1000 ml of ringer lactate/normal saline at the rate of 40-60 drops per minute	
		10.4.2	Continues uterine massage	
		10.4.3	If uterus is still relaxed, gives other Uterotonics as recommended	
		10.4.4	If uterus is still relaxed, performs mechanical compression in the form of bimanual uterine compression or external aortic compression or balloon tamponade ***(Ask doctor/nurse as per facility protocol)***	
		10.4.5	If uterus is still relaxed, refers to higher center while continuing mechanical compression	
10.5	Manages PPH due to retained placenta/placental bits	10.5.1	Identifies retained placenta if placenta is not delivered within 30 minutes of delivery of baby or the delivered placenta is not complete	
		10.5.2	Initiates 20 IU oxytocin drip in 1000 ml of ringer lactate/normal saline at the rate of 40-60 drops per minute	
		10.5.3	Refers to higher center if unable to manage	
		10.5.4	Performs Manual Removal of Placenta (MRP) ***(Ask Doctor)***	
**Standard 10 final response (Yes/No):**				
**11**	**Provider identifies and manages severe Pre-eclampsia/Eclampsia (PE/E)**			
11.1	Identifies mothers with severe PE/E	11.1.1	Dipsticks for proteinuria testing in labor room are available	
		11.1.2	Records BP at admission	

		11.1.3	Identifies danger signs or presence of convulsions	
11.2	Gives correct regimen of Inj. $MgSO_4$ for prevention and management of convulsions	11.2.1	MgSO4 in labor room (at least 20 ampoules) is available	
		11.2.2	Inj. MgSO4 is appropriately administered	
11.3	Facilitates prescription of anti-hypertensive	11.3.1	Antihypertensive are available	
		11.3.2	Facilitates prescription of anti-hypertensive	
11.4	Ensures specialist attention for care of mother and newborn	11.4.1	Ensures specialist attention for care of mother and newborn	
11.5	Performs nursing care	11.5.1	Performs nursing care	
**Standard 11 final response (Yes/No):**				
**12**	**Provider performs newborn resuscitation if baby does not cry immediately after birth**			
12.1	Performs steps for resuscitation within first 30 seconds	12.1.1	Suction equipment/mucus extractor is available	
		12.1.2	Shoulder roll is available	
		12.1.3	Performs following steps on mothers abdomen: dries the baby; immediate clamps and cuts the cord and shifts the baby to radiant warmer if still not breathing	
		12.1.4	Performs following steps under radiant warmer: Positioning, Suctioning, Stimulation, Repositioning (PSSR)	
12.2	Provider initiates bag and mask ventilation for 30 seconds if baby still not breathing	12.2.1	Functional ambu bag with mask for pre-term baby is available	
		12.2.2	Functional ambu bag with mask for term baby is available	

Supplement to: Delaney MM, Usmanova G, Nair TS, et al. Does quality certification work? An assessment of Manyata, a childbirth quality program in India's private sector. *Glob Health Sci Pract*. 2022;10(6):e2200093. https://doi.org/10.9745/GHSP-D-22-00093

		12.2.3	Initiates bag and mask ventilation using room air, If not breathing well –<br>- Applies appropriately sized mask correctly<br>- Gives 5 ventilatory breaths and looks for chest rise	
		12.2.4	If there is no chest rise after 5 breathes, takes corrective measures (Corrects the position / sucks mouth and nose / checks the seal / gives ventilation with increased pressure). If there is adequate chest rise, continues bag and mask ventilation for 30 seconds and reassess	
12.3	Provider takes appropriate action if baby doesn't respond to ambu bag ventilation after golden minute	12.3.1	Functional oxygen cylinder (with wrench) with new born mask is available	
		12.3.2	Functional stethoscope is available	
		12.3.3	Assesses breathing, if still not breathing continues bag and mask ventilation	
		12.3.4	Checks heart rate/cord pulsation	
		12.3.5	If heart rate is <100 / ≥ 100/ min and baby is still not breathing, continues bag and mask ventilation and connects oxygen. ***(Ask doctor/nurse as per facility protocol)***	
		12.3.6	If heart rate is >=100 and baby is breathing well or at any point, if baby starts breathing, provides observational care with mother ***(Ask doctor/nurse as per facility protocol)***	
		12.3.7	If baby is still not breathing and advance help is not available, then refers to higher center continuing bag and mask ventilation with oxygen ***(Ask doctor/nurse as per facility protocol)***	
**Standard 12 final response (Yes/No):**				

Supplement to: Delaney MM, Usmanova G, Nair TS, et al. Does quality certification work? An assessment of Manyata, a childbirth quality program in India's private sector. *Glob Health Sci Pract*. 2022;10(6):e2200093. https://doi.org/10.9745/GHSP-D-22-00093

13	**Provider ensures care of newborn with small size at birth**			
13.1	Preterm labour	13.1.1	Facility staff adheres to standard protocol for identification and management of preterm labour. Correctly estimates gestational age to confirm that labour is preterm.	
		13.1.2	Administration of corticosteroid for is ensured between 24-34 weeks.	
13.1	Facilitate specialist care in newborn weighing <1800 gm	13.1.1	Facilitates specialist care in newborn <1800 gm (refer to FBNC/seen by pediatrician)	
13.2	Facilitates assisted feeding whenever required	13.2.1	Facilitates assisted feeding whenever required	
13.3	Facilitates thermal management including kangaroo mother care	13.3.1	Facilitates thermal management including KMC	
**Standard 13 final response (Yes/No):**				
**Beyond Delivery:**				
14	**The facility adheres to universal infection prevention protocols**			
14.1	Instruments and re-usable items are adequately and appropriately processed after each use	14.1.1	Facilities for sterilization of instruments are available	
		14.1.2	Instruments are sterilized after each use	
		14.1.3	Delivery environment such as labor table, contaminated surfaces and floors are cleaned after each delivery	
14.2	Biomedical waste is segregated and disposed of as per the guidelines	14.2.1	Color coded bags for disposal of biomedical waste are available	
		14.2.2	Biomedical waste is segregated and disposed of as per the guidelines	

14.3	Performs hand hygiene before and after each procedure, and sterile gloves are worn during delivery and internal examination	14.3.1	Performs hand hygiene before and after each procedure, and sterile gloves are worn during delivery and internal examination	
14.4	PPE	14.4.1	Availability of Masks, caps and protective eye cover, sterile gloves, elbow length gloves, disposable gown/Apron, utility gloves for housekeeping staff.	
14.5	Infection control protocols	14.5.1	Separation of routes for clean and dirty items; Availability of disinfectant & cleaning agents, Standard practice of mopping and scrubbing are followed.	
14.6	Microbiological surveillance	14.6.1	Provision for Passive and active culture surveillance of critical & high risk areas. Microbiological surveillance : Swab are taken from infection prone surfaces such as delivery tables, door, handles, procedure lights etc	
14.7	Facilitates prevention of mother to child transmission of HIV	14.7.1	Facility staff adheres to standard protocols for Management of HIV in Pregnant Woman & Newborn.	
**Standard 14 final response (Yes/No):**				
**Postnatal Care Standard:**				
**15**	**Provider ensures adequate postpartum care package is offered to the mother and baby – at discharge**			
15.1	Conducts proper physical examination of mother and newborn during postpartum visits	15.1.1	Conducts mother's examination: breast, perineum for inflammation; status of episiotomy/tear suture; lochia; calf tenderness/redness/ swelling; abdomen for involution of uterus, tenderness or distension	
		15.1.2	Conducts newborn's examination: assesses feeding of baby; checks weight, temperature, respiration, color of skin and cord stump	

15.2	Identifies and appropriately manages maternal and neonatal sepsis	15.2.1	Checks mother's history related to maternal infection	
		15.2.2	Checks mother's temperature	
		15.2.3	Gives correct regimen of antibiotics**(*Ask doctor/nurse as per facility protocol)***	
		15.2.4	Checks baby's temperature and other looks for other signs of infections	
		15.2.5	Gives correct regime of antibiotics/refers for specialist care **(*Ask doctor/nurse as per facility protocol)***	
15.3	Correctly diagnoses postpartum depression based on history and symptoms	15.3.1	Provides emotional support and refers woman to specialist care	
15.4	Counsels on importance of exclusive breast feeding	15.4.1	Provides counselling and assistance on the importance of exclusive breast feeding and techniques of breast feeding	
15.5	Counsels on danger signs, postpartum family planning	15.5.1	Counsels on return of fertility and healthy timing and spacing of pregnancy – Counsels on postpartum family planning to mother at discharge	
**Standard 15 final response(Yes/No):**				
**Cesarean Delivery Standard: (Procedural steps are not mentioned)**				
**16**	**Provider reviews clinical practices related to cesarean delivery at regular intervals**			
16.1	Provider determines the need of cesarean delivery as per indication	16.1.1	Ensures all cesarean delivery cases are classified as per modified Robson's criteria (Annexure 1)	
		16.1.2	Obtains written informed consent from pregnant woman/her family for cesarean delivery and anesthesia	

Supplement to: Delaney MM, Usmanova G, Nair TS, et al. Does quality certification work? An assessment of Manyata, a childbirth quality program in India's private sector. *Glob Health Sci Pract*. 2022;10(6):e2200093. https://doi.org/10.9745/GHSP-D-22-00093

16.2	Operation theatre is adequately equipped for conducting cesarean delivery	16.2.1	Number of OT tables in the OT are appropriate as per the cesarean delivery load (Annexure 2)	
		16.2.2	Adequate supplies and equipment are available in the OT for cesarean delivery (Annexure 2)	
		16.2.3	Anesthesia tray with functional Boyle's apparatus is available	
		16.2.4	OT has adequate lighting, ventilation and temperature control	
		16.2.5	OT complex has provision for separate washing area with 24-hour running water supply and soap	
		16.2.6	OT complex has functional toilet and staff resting/changing area	
		16.2.7	Functional newborn care area is available in the OT	
		16.2.8	Adequate supplies and equipment are available for conducting adult/newborn resuscitation (Annexure 2)	
16.3	Reviews cesarean delivery cases through a clinical audit once every quarter	16.3.1	Reviews cesarean delivery cases through a clinical audit with team once every quarter in facility	
		16.3.2	Ensures that rate of complications of cesarean deliveries are periodically monitored in facility	
**Standard 16 final response(Yes/No):**				

Supplement to: Delaney MM, Usmanova G, Nair TS, et al. Does quality certification work? An assessment of Manyata, a childbirth quality program in India's private sector. *Glob Health Sci Pract*. 2022;10(6):e2200093. https://doi.org/10.9745/GHSP-D-22-00093

Supplement 2. Knowledge and Skills Assessments by State

Knowledge and skills assessments	OVERALL<br>Total N<br>Average Prescore (95% CI)<br>Average Postscore (95% CI)	JHARKHAND<br>Total N<br>Average Prescore (95% CI)<br>Average Postscore (95% CI)	MAHARASHTRA<br>Total N<br>Average Prescore (95% CI)<br>Average Postscore (95% CI)	UTTAR PRADESH<br>Total N<br>Average Prescore (95% CI)<br>Average Postscore (95% CI)
KNOWLEDGE ASSESSMENT				
Knowledge test scores<br>(Max score 20)	912<br>6.34 (6.14, 6.54)<br>13.17 (12.92, 13.41)	91<br>3.11 (2.42, 3.8)<br>11.12 (10.55, 11.7)	483<br>7.12 (6.86, 7.38)<br>11.18 (10.95, 11.4)	338<br>6.11 (5.82, 6.4)<br>16.56 (16.24, 16.87)
OSCE ASSESSMENT				
Overall<br>(Max score 46)	878<br>7.97 (7.60, 8.33)<br>34.33 (34.05, 34.60)	82<br>7.66 (6.47, 8.84)<br>33.98 (33.17, 34.78)	478<br>8.86 (8.39, 9.33)<br>34.34 (33.95, 34.73)	318<br>6.7 (6.05, 7.34)<br>34.39 (33.96, 34.83)
AMSTL<br>(Max Score=10; Passed=8 or Higher)	888<br>1.53 (1.41, 1.65)<br>9.12 (9.05, 9.18)	82<br>1.93 (1.54, 2.31)<br>8.43 (8.27, 8.59)	488<br>1.62 (1.46, 1.78)<br>9.15 (9.05, 9.25)	318<br>1.29 (1.09, 1.49)<br>9.25 (9.16, 9.34)
Newborn Resuscitation<br>(Max Score=16; Passed=12 or Higher)	890<br>1.37 (1.26, 1.48)<br>10.53 (10.43, 10.63)	82<br>1.39 (0.91, 1.87)<br>11.27 (10.99, 11.55)	490<br>1.27 (1.13, 1.4)<br>11.03 (10.92, 11.14)	318<br>1.53 (1.35, 1.72)<br>9.57 (9.41, 9.72)
Antenatal Complications<br>(Max Score=8; Passed=6 or Higher)	882<br>1.32 (1.22, 1.42)<br>6.13 (6.01, 6.25)	82<br>1.49 (1.18, 1.79)<br>6.15 (5.99, 6.3)	482<br>1.33 (1.19, 1.46)<br>5.66 (5.48, 5.84)	318<br>1.26 (1.08, 1.44)<br>6.84 (6.72, 6.96)
Postnatal Complications<br>(Max Score=12; Passed=9 or Higher)	880<br>3.76 (3.55, 3.97)<br>8.53 (8.37, 8.68)	82<br>2.85 (2.03, 3.68)<br>8.13 (7.61, 8.66)	480<br>4.67 (4.4, 4.93)<br>8.47 (8.28, 8.66)	318<br>2.61 (2.29, 2.94)<br>8.71 (8.44, 8.99)

Supplement to: Delaney MM, Usmanova G, Nair TS, et al. Does quality certification work? An assessment of Manyata, a childbirth quality program in India's private sector. *Glob Health Sci Pract*. 2022;10(6):e2200093. https://doi.org/10.9745/GHSP-D-22-00093

Supplement 3. Knowledge and Skills Assessments by Facility Size

Knowledge and skills assessments	OVERALL<br>Total N<br>Average Pre Score (95% CI)<br>Average Post Score (95% CI)	SMALL FACILITIES (1 to 20 beds)<br>Total N<br>Average Pre Score (95% CI)<br>Average Post Score (95% CI)	MEDIUM FACILITIES (21 to 50 beds)<br>Total N<br>Average Pre Score (95% CI)<br>Average Post Score (95% CI)	LARGE FACILITIES (51+ beds)<br>Total N<br>Average Pre Score (95% CI)<br>Average Post Score (95% CI)
KNOWLEDGE ASSESSMENT				
Knowledge test scores<br>(Max score 20)	912<br>6.34 (6.14, 6.54)<br>13.17 (12.92, 13.41)	486<br>6.63 (6.37, 6.89)<br>12.31 (12.00, 12.61)	281<br>5.81 (5.42, 6.20)<br>14.24 (13.77, 14.71)	96<br>6.36 (5.67, 7.05)<br>14.98 (14.23, 15.73)
OSCE ASSESSMENT				
Overall<br>(Max score 46)	878<br>7.97 (7.60, 8.33)<br>34.33 (34.05, 34.60)	474<br>8.09 (7.63, 8.55)<br>33.60 (33.21, 33.99)	262<br>8.09 (7.29, 8.89)<br>35.31 (34.85, 35.77)	93<br>7.17 (5.98, 8.37)<br>34.78 (33.94, 35.62)
AMSTL<br>(Max Score=10; Passed=8 or Higher)	888<br>1.53 (1.41, 1.65)<br>9.12 (9.05, 9.18)	481<br>1.60 (1.44, 1.76)<br>9.01 (8.91, 9.11)	263<br>1.58 (1.35, 1.81)<br>9.21 (9.10, 9.31)	95<br>1.41 (1.06, 1.76)<br>9.31 (9.13, 9.48)
Newborn Resuscitation<br>(Max Score=16; Passed=12 or Higher)	890<br>1.37 (1.26, 1.48)<br>10.53 (10.43, 10.63)	482<br>1.26 (1.13, 1.39)<br>10.57 (10.44, 10.70)	264<br>1.56 (1.32, 1.81)<br>10.44 (10.24, 10.64)	95<br>1.71 (1.34, 2.07)<br>10.42 (10.14, 10.71)
Antenatal Complications<br>(Max Score=8; Passed=6 or Higher)	882<br>1.32 (1.22, 1.42)<br>6.13 (6.01, 6.25)	476<br>1.26 (1.13, 1.39)<br>5.73 (5.55, 5.90)	263<br>1.50 (1.29, 1.70)<br>6.64 (6.48, 6.80)	94<br>1.51 (1.17, 1.85)<br>6.53 (6.27, 6.79)
Postnatal Complications<br>(Max Score=12; Passed=9 or Higher)	880<br>3.76 (3.55, 3.97)<br>8.53 (8.37, 8.68)	475<br>3.98 (3.70, 4.26)<br>8.28 (8.07, 8.49)	263<br>3.48 (3.08, 3.89)<br>8.97 (8.72, 9.22)	93<br>2.51 (1.88, 3.13)<br>8.53 (7.97, 9.08)
